# Localization and expression dynamics of an RNA Pol I core subunit in response to fasting in *C. elegans*

**DOI:** 10.17912/micropub.biology.001472

**Published:** 2025-02-04

**Authors:** Nada Al-Refaie, Francesco Padovani, Kurt M. Schmoller, Daphne S. Cabianca

**Affiliations:** 1 Institute of Functional Epigenetics, Helmholtz Zentrum München, Munich, Bavaria, Germany; 2 Faculty of Medicine, Ludwig-Maximilians-Universität München, Munich, Bavaria, Germany

## Abstract

Nutrient availability influences ribosome biogenesis, requiring dynamic regulation of RNA Pol I activity. In *C. elegans* , fasting reduces pre-rRNA levels. However, whether this reduction stems from a regulation of RNA Pol I expression remains unclear. Here, we examined how the nutritional status affects the localization and expression levels of RPOA-2 , a core subunit of RNA Pol I, in the intestine. We found that RPOA-2 retains its nucleolar localization regardless of animals being fed, fasted or fed after fasting. Interestingly, fasting reduces RPOA-2 protein amounts which are restored upon feeding. These findings suggest that the availability of RNA Pol I core subunits contributes to the regulation of rDNA transcription in response to nutrients.

**Figure 1. RPOA-2 localization and levels in fed, fasted and fed after fasting conditions in the C. elegans intestine:**
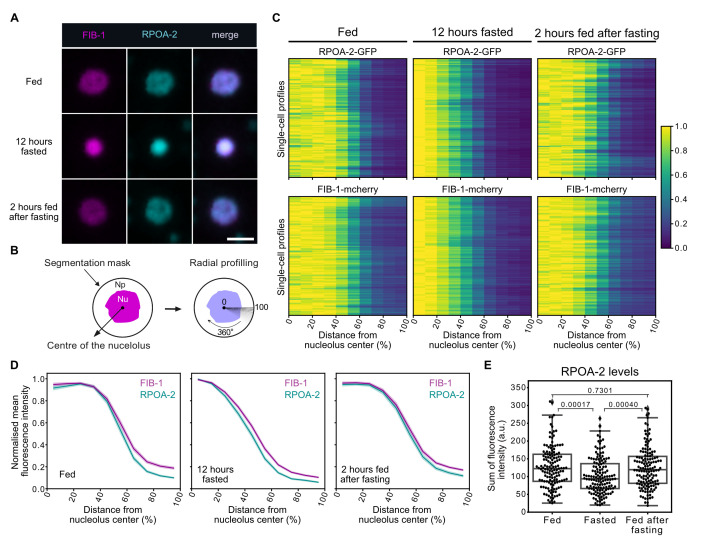
**A.** Single focal planes of representative intestinal nuclei expressing RPOA-2-GFP and FIB-1-mCherry in wild-type (wt) L1 larvae that were continuously fed, 12 hours fasted, or fed for 2 hours after fasting. Scale bar, 2 μm. **B.** Schematic representation of the quantification approach for RPOA-2 and FIB-1 signal distribution. A segmentation mask was generated around the nucleolus and the surrounding nucleoplasm based on the FIB-1-mCherry signal. Radial fluorescence intensity profiles for RPOA-2-GFP and FIB-1-mCherry were extracted from the nucleolar center (0) to the edge of the segmented mask (100) along all possible angles. The radial profiles were then averaged to obtain the single-cell fluorescence profile, which is displayed in each row of the heatmap in (C). Np, nucleoplasm; Nu, nucleolus. **C.** Heatmaps showing the quantification of radial fluorescence intensity profiles of RPOA-2-GFP and FIB-1-mCherry as described in (B) in intestinal cells of wt animals fed, fasted and fed after fasting. 143-144 intestinal cells were analyzed for each condition from 3 independent biological replicates. Each row corresponds to a single cell. **D.** Line plots of the averaged single-cell profiles shown in (C). The shaded area represents the 95% confidence interval of the mean profile. **E.** Boxplot showing the levels of RPOA-2-GFP in the intestinal cells of fed, 12 hours fasted and 2 hours-fed animals after fasting. 141-143 cells were analyzed for each condition from 3 independent biological replicates. Box limits are 25th and 75th percentiles, whiskers denote 1.5 times the interquartile ranges, points outside the whiskers are outliers, and the median is shown as a line. P-values from two-sided Wilcoxon rank sum tests are indicated.

## Description

Cells adapt their protein production capacity to fluctuations in nutrient availability by adjusting ribosome concentration. Transcription of ribosomal RNA (rRNA) by RNA polymerase I (RNA Pol I) is a key regulatory step in controlling ribosome biogenesis (Laferté et al., 2006) . RNA Pol I acts within the nucleolus, where also rRNA maturation and ribosome subunit assembly occur. Consequently, the nucleolus is remodelled in response to changes in nutrient availability (Tiku et al., 2017) . In yeast, nutrient starvation leads to a drastic decrease of nucleolar size, accompanied by the dissociation of RNA Pol I core subunits from the rDNA and their release from the nucleolus into the nucleoplasm (Tsang et al., 2003) . Similarly, our recent work shows that in response to fasting of *C. elegans* , nucleolar size is drastically reduced and pre-rRNA levels are strongly diminished (Al-Refaie et al., 2024) . However, whether RNA Pol I localization is also affected remains unexplored. To address this, we used animals expressing RPOA-2 , a core subunit of RNA Pol I (homologous to human POLR1B and yeast Rpa2), fused to GFP through CRISPR-Cas9-mediated editing of its endogenous locus. Unlike animals lacking RPOA-2 (Zhao et al., 2023, Liao et al., 2021) , RPOA-2 tagged worms develop normally, indicating that tagging does not significantly impair RPOA-2 functionality. Our previous findings showed that the volume of the nucleolus is more strongly reduced in the intestine compared to other tissues (Al Refaie et al., 2024) . Thus, we decided to monitor RPOA-2 localization relative to nucleoli, visualized using FIB-1 /Fibrillarin-mCherry, in intestinal cells of L1 larvae under continuously fed, 12-hour fasted, and fed for 2 hours after fasting conditions. We found that RPOA-2 consistently remained located inside the nucleolus in all conditions ( Figure 1A ). To quantify this observation, we measured the fluorescence intensity of RPOA-2 and FIB-1 from the nucleolar center to the surrounding nucleoplasm across all possible angles as depicted in Figure 1B . The radial fluorescence intensity profiles were then averaged to generate a single, averaged signal profile for each nucleus ( Figure 1C ). RPOA-2 remained localized within the nucleolus in fed and fasted conditions as well as in animals fed after fasting, with RPOA-2 being slightly less enriched at the nucleolar center in fed animals compared to fasted and fed post-fasting conditions ( Figure 1C-D ). While we cannot rule out the possibility that other tissues respond differently than the intestine, these findings indicate that the relocation of RNA Pol I subunits away from the nucleolus is not a universal feature of its inhibition by nutrient deprivation.

A recent study showed that the expression of POLR1E, an RNA Pol I subunit, is upregulated in response to increased serum concentration in mammalian cells (Theophanous et al., 2023) . Thus, we decided to test whether fasting alters RPOA-2 expression levels using the same experimental setup as described above. We found that RPOA-2 protein levels are moderately reduced in intestinal cells of fasted animals but increase again upon feeding, reaching levels comparable to those in animals that were continuously fed without exposure to fasting ( Figure 1E ). Whether this effect is intestine-specific or occurs more broadly remains to be determined. While RNA Pol I transcription is primarily regulated by specific transcription factors and the chromatin state of the rDNA region (Goodfellow et al., 2013) , the modulation of core RNA Pol I subunit levels may also contribute to rDNA transcription regulation. Furthermore, having only a modest reduction in subunit amounts during fasting might allow for a rapid reinitiation of rDNA transcription once food becomes available.

## Methods

**Strain used in this study:**

DCW325 qzIs15[rpoa-2p::degron1(TIR1)::GFP::rpoa-2] I, wrdSi23 [eft-3p::TIR1::F2A::BFP::AID*::NLS::tbb-2 3'UTR] I, mCherry::FIB-1 (ustIS36) II

**Feeding, fasting and feeding fasted worms**

Worms were maintained well-fed on *Escherichia coli* OP50 bacteria on nematode growth medium (NGM) agar plates at 20 °C for at least two generations.

Fed L1 larvae (L1s) were obtained by washing plates of mixed-stage animals twice with M9 buffer to remove adults and larvae. The washes were performed with gentle swirling to avoid removing the bacteria. To obtain synchronized L1s, the embryos remaining on the plate were allowed to hatch for 2 hours. Afterwards, they were collected for imaging.

For fasted L1s, embryos were isolated by standard hypochlorite treatment and maintained in M9 buffer on a roller at 20 °C for 24 hours. Then, they were prepared for imaging. L1s hatch approximately 12 hours after hypochlorite treatment (Webster et al., 2022 and our own observation), and hence, this timepoint was considered 0 h of fasting. Consequently, 12 hours of fasting corresponds to 24 hours after hypochlorite treatment.

Feeding fasted worms was performed by placing 12 hours-fasted L1s on OP50-seeded NGM plates for 2 hours, after which they were collected for imaging.

**Microscopy**

Microscopy was carried out using a live-cell imaging system (Confocal Spinning Disk Microscope) from Visitron Systems GmbH, equipped as follows: Nikon Eclipse Ti2 microscope with a Plan apo λ 100×/1.45 oil objective, Yokogawa CSU-W1 confocal scanner unit, VS-Homogenizer, Electron Multiplying CCD camera (Andor iXon Series) and VisiView software for acquisition.

Live microscopy was carried out on 2% agarose pads supplemented with 0.15% sodium azide (Interchim) to paralyse the worms, as previously described (Al-Refaie et al., 2024) . All Images were acquired with the Plan apo λ 100×/1.45 oil objective. For each image, 70-75 stacks were captured with a *z* -spacing of 200 nm.

**Image analysis**

*RPOA-2 expression quantification*

The RPOA-2-GFP signal was quantified using the SpotMAX software (Padovani et al., 2024) . The analysis steps were as follows: (1) Area around each RPOA-2-GFP signal in the mid intestine were manually segmented in 2D at the central plane of the signal using Cell-ACDC (Padovani et al., 2022) . (2) The segmentation mask was extended 8 slices (of 200 nm each) above and below the central plane to cover the whole signal. (3) Application of a 3D Gaussian filter with a small sigma (0.75 voxels). (4) Instance 3D segmentation of the signal using the best-suited automatic thresholding algorithm (Li algorithms from the Python library scikit-image). (5) The background-corrected sum of all the voxel values belonging to each segmented signal was calculated. Background correction was performed by subtracting the median intensity of the pixels inside the manually segmented area at the central *z* -slice but outside of the segmented RPOA-2 signal.

*Radial fluorescence intensity profiling of RPOA-2-GFP and FIB-1-mCherry*

Based on the FIB-1 /fibrillarin signal, each nucleolus and the surrounding nucleoplasm in the mid-intestine was manually segmented in 2D at its central plane, followed by manual annotation of the nucleolar center using Cell-ACDC (Padovani et al., 2022) . To quantify the signal distribution of RPOA-2 and FIB-1 , we implemented a custom routine written in Python. The analysis steps were as follows: (1) Determination of the segmented area contours (using the function from OpenCV package called ‘findContours‘). (2) Extraction of the intensity profiles from the centre to all the points on the contour. (3) Normalization of the distance for each profile from the centre to the contour point (0% centre, 100% contour). (4) Binning the intensities into 10% width bins (for example, intensities at any distance in the ranges 0–10%, 10–20%, 20–30% etc. were considered to be at 5%, 15%, 25%, etc. (bin centre) distance from the centre). (5) Averaging of the binned normalized profiles along the distance to obtain single-cell average profiles. (6) Normalization of each single-cell profile by its max intensity value. (7) Averaging and standard error calculation of the single-cell profiles within the same experimental conditions to obtain single-condition average profiles and its associated standard errors.
